# Optimization of polycaprolactone - based nanofiber matrices for the cultivation of corneal endothelial cells

**DOI:** 10.1038/s41598-021-98426-6

**Published:** 2021-09-22

**Authors:** Marcus Himmler, Fabian Garreis, Friedrich Paulsen, Dirk W. Schubert, Thomas A. Fuchsluger

**Affiliations:** 1 Department of Ophthalmology, University Medical Center Rostock, Doberaner Str. 140, 18057 Rostock, Germany; 2 Institute of Polymer Materials, Friedrich-Alexander University Erlangen-Nuremberg, Martenstr. 7, 91058 Erlangen, Germany; 3 Institute of Functional and Clinical Anatomy, Friedrich-Alexander University Erlangen-Nuremberg, Universitaetsstr. 19, 91054 Erlangen, Germany; 4 Department of Operative Surgery and Topographic Anatomy, Sechenov University, Trubetskaya Str. 8-2, Moscow, Russian Federation 119991

**Keywords:** Bioinspired materials, Biomaterials - cells, Biomedical materials, Tissues, Implants, Tissue engineering and regenerative medicine

## Abstract

Posterior lamellar transplantation of the eye’ s cornea (DSAEK, DMEK) currently is the gold standard for treating patients with corneal endothelial cell and back surface pathologies resulting in functional impairment. An artificial biomimetic graft carrying human corneal endothelium could minimize the dependency on human donor corneas giving access to this vision-restoring surgery to large numbers of patients, thus reducing current long waiting lists. In this study, four groups of electrospun nanofibrous scaffolds were compared: polycaprolactone (PCL), PCL/collagen, PCL/gelatin and PCL/chitosan. Each of the scaffolds were tissue-engineered with human corneal endothelial cells (HCEC-B4G12) and analyzed with regard to their potential application as artificial posterior lamellar grafts. Staining with ZO-1 and Na^+^/K^+^-ATPase antibodies revealed intact cell functionalities. It could be shown, that blending leads to decreasing contact angle, whereby a heterogeneous blend morphology could be revealed. Scaffold cytocompatibility could be confirmed for all groups via live/dead staining, whereby a significant higher cell viability could be observed for the collagen and gelatine blended matrices with 97 ± 3% and 98 ± 2% living cells respectively. TEM images show the superficial anchoring of the HCECs onto the scaffolds. This work emphasizes the benefit of blended PCL nanofibrous scaffolds for corneal endothelial keratoplasty.

## Introduction

Transparency of the cornea, the window to the eye, is a key feature for clear vision. The cornea has a thickness of about 550 µm and consists of five major layers, each contributing to maintain its functionality. The outermost layer is the corneal epithelium, a multilayer of epithelial cells. Besides the tear film, these cells are the first barrier against infections and highly reproducible, the epithelial cell layer is renewed every seven days^1^. The cells’ basement membrane rests on the Bowman’s membrane. It is separating the epithelial cell layer from the corneal stroma, the main layer of a cornea, consisting of highly aligned collagen fibrils. Towards the back surface of the cornea, towards the anterior chamber, the stroma is neighboured by the Descemet’s membrane supporting a monolayer of evenly hexagonally shaped endothelial cells^1–4^.

The Descemet’s membrane of an adult is approximately 11.7 mm in circular diameter and the thickness can accumulate up to 10 µm with age^1,4^. Human corneal endothelial cells (HCEC) arrange in a highly ordered hexagonally shaped monolayer on top of the Descemet’s membrane. Single cells are about 18–20 µm in width and 5 µm in thickness^5^. In addition to adhesive contacts and gap junctions, the cells also have tight junctions, which means that the endothelium forms a diffusion barrier to the directly adjacent aqueous humour that helps to maintain the intraocular pressure of the anterior chamber. In the pump-leak mechanism, optimal hydration of the stroma with around 78% water content is maintained^1^. Hereby the leaking of the aqueous humour into the stroma is simultaneously balanced by the corneal endotheliums’ pump function (Na^+^/K^+^-ATPase, aquaporin 1 water channels), which removes excess fluid from the stroma thus ensuring transparency^1–3^. While new-borns have an endothelial cell density (ECD) around 6000 cells per mm^2^ the ECD decreases by apoptotic processes over the life span to about 2000–2500 cells per mm^2^ in the elderly^6^. However, there is a minimum threshold of 500–600 cells per mm^2^ at which the endothelial layer can maintain its draining function^3,6,7^. Even though the cells barely proliferate and despite apoptosis-related cell loss, migration and enlargement of cells ensures a continuously closed monolayer. Thereby, the cells lose their homogenous morphology and arrange in a rather odd-shaped way^1^. Due to disease or surgery, the ECD may significantly fall below this lower limit and the hydrophilic stroma fills up with aqueous humour. Water uptake into the stroma impairs the regular arrangement of the collagen fibrils, subsequent stromal swelling impairs optic properties of the cornea which clinically shows haziness, ultimately results in blindness of the patient due to an opaque cornea^1,4^.Treatments include the transplantation of the whole cornea (penetrating keratoplasty, PK) or rather of single corneal layers, so-called lamellar (endothelial) keratoplasty^8^. Descemet’s membrane endothelial keratoplasty (DMEK) is the current gold standard in treating patients with a corneal endothelial cell pathology^9–11^. DMEK describes isolated Descemet’s membrane plus corneal endothelial cell transplantation. Current therapies rely on the availability of human donor tissue. To date the prime indication for corneal transplantation is an isolated back surface endothelial cell disease, Fuchs endothelial corneal dystrophy (about 39% of all corneal transplantations). Importantly, merely one out of 70 globally requested donor corneas could be served and over 50% of the world’s population has no access to corneal transplantation^12^. The limited availability of donor corneas is further reduced as particular tissue requirements are mandatory. Due to the limited proliferative capacity of endothelial cells, a minimum donor ECD of 2000 cells per mm^2^ is required to minimize the risk of primary graft failure^12–14^. Different approaches have been made to overcome this shortage and to restore the endothelial cell monolayer, for example using cell therapies^6,15^. Nevertheless, there is still a major dependency on donor corneas. Another approach is the development of synthetic, artificial biomimetic grafts^16–24^ or of biological derived grafts^25–40^. Biologically-derived grafts harbour risks of contamination and inconsistencies concerning physical properties as transparency or mechanical strength. By contrast, synthetic derived grafts offer the possibility for scaffolds with defined and constant properties, adjustable characteristics and the potential to integrate drugs. In comparison to films or hydrogels, nanofibrous matrices mimic the native tissue. In this context, electrospun nanofibrous scaffolds, resembling the fibrous structure of the natural Descemet’s membrane seem to be a promising approach towards artificial posterior lamellar grafts to be used in DMEK surgeries.

In this study, nanofiber scaffolds were produced using electrospinning, as demonstrated by others a useful method for tissue engineering^41^. Their highly porous fibrous network provides a permeable but solid scaffold, mimicking the Descemet’s membrane^5^. Poly(ε-caprolactone) (PCL) is a versatile polymer for biomedical applications as approved by the Food and Drug Administration (FDA) for implants in vivo^42^ and has already proven to be a promising scaffold material for corneal endothelial cells^16^. Due to its hydrophobicity, PCL is often blended with natural polymers to improve the biocompatibility^43–47^. In this study, collagen and gelatine as mammal derived proteins as well as chitosan, known for its antimicrobial efficacy were used^48^. PCL was blended with collagen, gelatine and chitosan and the resulting scaffolds were examined for fiber morphology, scaffold characteristics and biocompatibility.

## Results

### Production and analysis of electrospun matrices

Electrospinning offers the possibility to produce nanoscaled fibers from polymer solutions or from polymer melts. The process is well described and can be found in literature e.g. Wendorff et al.^49^. Therefore, polymer solutions were prepared following the data in Table 1.

**Table 1:** Composition of the different scaffold materials.

Material	Solvent system	Polymer concentration	Voltage (kV)	Distance (cm)	Flow rate (ml l^−1^)
PCL-1	chloroform/ethanol (7/3)	0.12 g/ml	20	22	1
PCL-2	formic acid/acetic acid (7/3)	0.12 g/ml	15	17	0.2
PCL-COL	90% acetic acid	0.08 g/ml PCL, 0.4 g/ml COL	15	15	1
PCL-GEL	90% acetic acid	0.08 g/ml PCL, 0.4 g/ml GEL	15	15	0.2
PCL-CHI	formic acid/acetic acid (7/3)	0.114 g/ml PCL, 0.06 g/ml CHI	22	17	0.2

Electrospinning was performed on pure PCL from different solvent systems, resulting in various fiber diameters and on PCL-blends with collagen, gelatine and chitosan. Corresponding electrospinning parameters are given.

PCL (M_W_ = 80,000 g mol^−1^, Sigma Aldrich, Saint Louis, MO, USA) was diluted in both solvent systems chloroform/ethanol (ratio 7/3, PCL-1) and formic acid/acetic acid (ratio 7/3, PCL-2), forming solutions of different viscosities and therefore of different fiber diameters. Collagen type I was purchased from Symathese (Lyon, France). Gelatine (type A, porcine skin) and chitosan (low molecular weight) were purchased from Sigma Aldrich (Saint Louis, MO, USA). Depending on the characteristics of the solution, voltage, needle-to-collector distance and flow rate was selected to achieve a homogenous and bead-free fiber morphology. For the chitosan-blended solution, only a very low content could be diluted as it is a highly hygroscopic polysaccharide with a huge impact on the viscosity of the spinning solution. Thus, the resulting fibers from the PCL-CHI solution consist of only 5% chitosan by weight. In comparison, PCL blended with collagen or gelatine consists of 33% blend phase by weight. The spinning time was set to produce scaffolds with a sufficient thickness for the subsequent experiments. Thickness of resulting scaffolds was in the range from 10 to 60 µm. The protocols for the solutions and the corresponding electrospinning parameters were previously published by our group^50,51^. PCL-GEL was adapted from the electrospinning of PCL-COL from Dippold et al.^50^. Solutions were stirred with 300 rpm for no longer than 4 h at ambient conditions prior to the spinning process.

**Figure 1:**
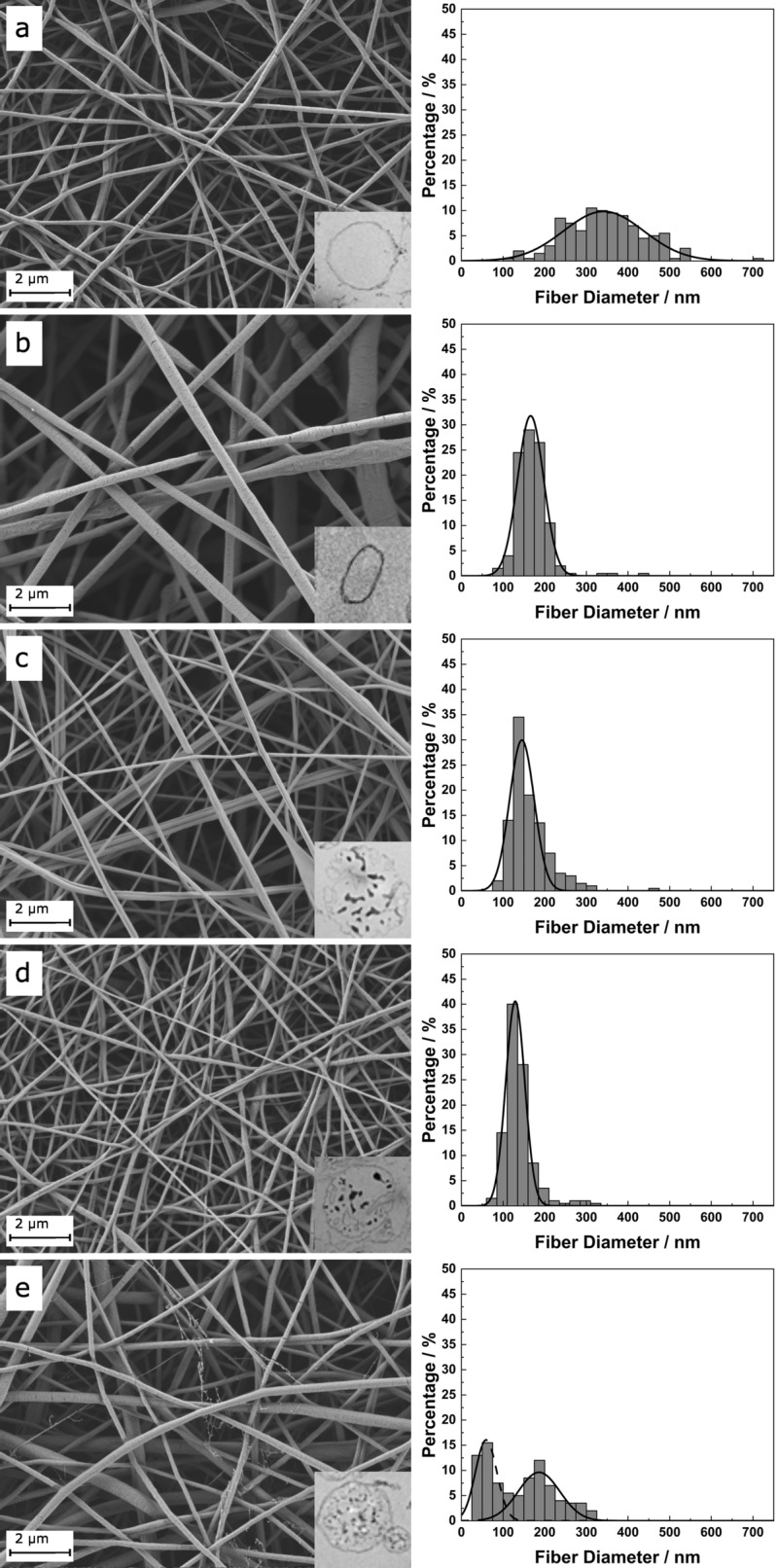
SEM images and corresponding fiber diameter distribution histograms. Insets show exemplary TEM images (0.25 µm in width and height) of the fiber cross sections: (**A**) PCL-1; (**B**) PCL-2; (**C**) PCL-COL; (**D**) PCL-GEL; (**E**) PCL-CHI. Fiber diameters from A-D are following a Gaussian distribution. In the case of PCL-CHI a bimodal distribution is observed.

In Fig. 1 the SEM images as well as the corresponding fiber diameter histograms are shown. From the solvent system of chloroform and ethanol (PCL-1, Fig. 1a) smooth, circular fibers were fabricated. The average diameter was 395 ± 226 nm. As shown in the corresponding histogram, the fiber diameter distribution was rather broad and indicates the so-called jet splitting and quantization as recently theoretically described^52^. In comparison, electrospinning of PCL from acetic and formic acid (PCL-2) resulted in significantly smaller fiber diameters (Fig. 1b) with an average of 169 ± 39 nm. Electrospinning of PCL-COL and PCL-GEL solutions (Fig. 1c and d) resulted in average fiber diameter of 162 ± 48 nm and 137 ± 37 nm, respectively. Electrospinning of PCL-CHI produced two regimes of fiber diameter, significantly different from each other as shown in Fig. 1e. The scaffold consisted of a network of fibers with an average fiber diameter of 186 nm. Between these, thin fibers with an average fiber diameter of 59 nm were spun. Overall, an average fiber diameter of 174 ± 119 nm was obtained. The resulting fiber diameters and thus pore geometries were sufficiently small that in the following cell cultivation cells attached on top of the scaffold rather than growing into the pores.

The insets in Fig. 1 show the cross sections of the fibers from TEM imaging. Fibers from solely PCL revealed a homogenous cross section without any precipitations. In contrast, the cross section of all blend scaffolds showed an inhomogeneous cross section with precipitations due to the staining of organic material.

**Figure 2:**
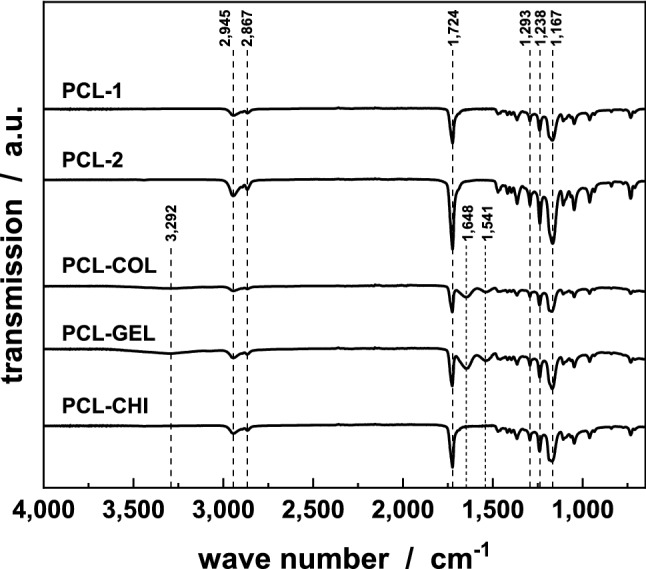
FT-IR spectra of all samples. Significant peaks for collagen and gelatine are shown, indicating a successful integration of the blend components in the PCL matrix. For the chitosan-blended nanofibers, the typical peak at 3.292 cm^−1^ is very weak due to the low content of chitosan in the initial spinning solution.

Infrared spectra of all scaffolds were recorded to verify the integration of the blend phases into the PCL matrix and are shown in Fig. 2. The IR spectrum of all five different scaffolds showed typical peaks for PCL independent of the solution system or blend components^53^. A signal at 2.945 cm^−1^ could be assigned to the asymmetric stretching of the CH_2_ and at 2.867 cm^−1^ the symmetric stretching of the CH_2_ could be detected. Further characteristic peaks were at 1.724 cm^−1^ (carbonyl stretching), 1.293 cm^−1^ (C–O and C–C stretching), 1.238 cm^−1^ (asymmetric C–O–C stretching) and at 1.170 cm^−1^ (symmetric C–O–C stretching). These explicit peaks could be found in all blended scaffolds. Beside the typical PCL peaks, PCL-COL and PCL-GEL scaffolds showed similar absorption bands. Both blends expressed characteristic peaks at 3.292 cm^−1^ that resembles to the stretching vibration of the N–H group. The characteristic peaks for the amide I, II, and III band could be found around 1.652 cm^−1^, 1.545 cm^−1^ and 1.240 cm^−1^, respectively. Both FT-IR spectra showed the integration of the blend phase in the PCL matrix. For the PCL-CHI scaffold, the characteristic peak for the N–H stretching of the amide bond was barely visible, possibly due to the small amount of chitosan in the initial spinning solution.

PCL is known for its relatively hydrophobic behaviour and therefore blending of PCL was considered to enhance the biocompatibility of the produced nanofibers. For pure PCL the measurements of film casted samples revealed a contact angle of 83.9° ± 3.8°. Blending with collagen or gelatine reduced the contact angle to 70.7° ± 4.8° and 57.2° ± 6.5°, respectively. The measurement of the contact angle revealed the advantageous influence of blending the PCL with collagen or gelatine. For both measurements a significant (*p* < 0.001) reduced contact angle could be detected. In the case of PCL-GEL film cast samples the contact angle was reduced by almost one third compared to the pure PCL film cast. Chitosan as blend component caused no significant change in contact angle (*p* > 0.05) with 80.7° ± 2.7°.

### Cell-scaffold interactions

**Figure 3:**
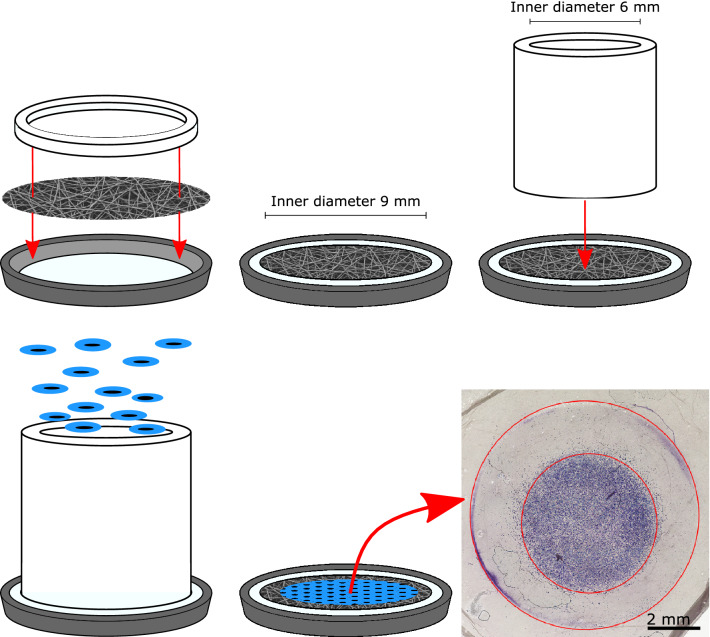
Fixation of the scaffold in the tissue carrier. The nanofibrous scaffold is fixed between the two rings. For the initial cell seeding, a sterile hollow cylinder is placed on the scaffold to enhance a precise cell attachment. HE staining of HCECs can be seen at the bottom right, displaying the cell seeding pattern resulting in a homogenous ECD within the inner red circle. The outer red circle indicates the circumference of the exposed sample.

In order to evaluate the HCEC’s proliferation capacity, a consistent number of cells need to be seeded for the conducted experiments. Therefore, a stable and reproducible method for cell seeding was developed, as shown schematically in Fig. 3. For the initial cell seeding, small PTFE cylinder with an inner diameter of 6 mm and an outer diameter slightly smaller than the inner diameter of the tissue carrier with a height of 10 mm were placed directly on the scaffold. Thus, the amount of cell suspension possible to use for the initial cell seeding could be increased and the cells were seeded on a precise area. This enables a homogeneous distribution of the cells on the scaffolds and thus a uniform initial cell density. After the cell cultivation, scaffolds could be removed for further analysis.

Due to the scaffold thickness and resulting opacity, cells cannot be displayed using conventional transmitted light microscopes and a subsequent staining of the cells for the evaluation of the cell density is necessary. In Fig. 3, bottom right, a picture of a scaffold with cells stained with hematoxylin and eosin (HE) is shown. The inner red circle displays the area where the cells were seeded and the outer red line indicates the edge of the previously fixed scaffold. The resulting scaffold outside the outer red circle was clamped in the tissue carrier and therefore not exposed to any medium while cell cultivation.

**Figure 4:**
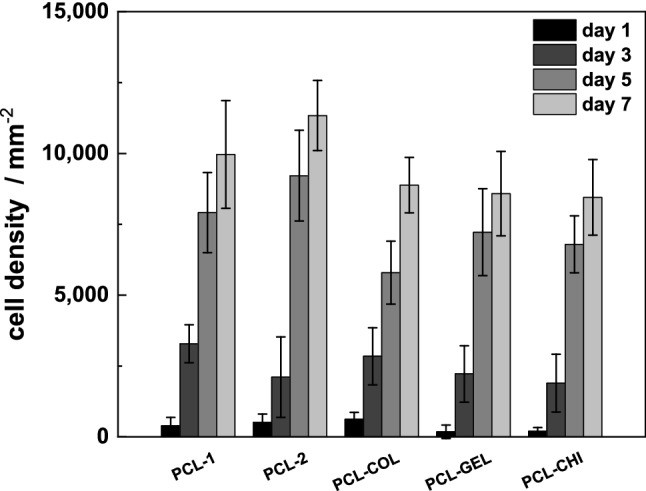
ECD for the different samples over an observation period of 7 days. Cells were seeded initially with an ECD of 700 cells per mm^2^.

In Fig. 4, the ECD of all samples for seven consecutive days is shown. After cell seeding at day 0, the measured ECD at day 1 was below the calculated initial cell density of 700 cells per mm^2^. This may indicate that less cells were initially seeded than calculated based on the cell suspension after trypsination and dilution. For all groups, a major increase in ECD could be found between day 3 and 5. The ECD on day 3 was in the range of 1.900 ± 1,020 cells per mm^2^ (PCL-CHI) and 3.200 ± 670 cells per mm^2^ (PCL-1), corresponding to the ECD in human corneal endothelium^1,3,4^. Further culture of the cells on the scaffolds lead to further increasing cell densities, whereby the cells were no longer present in a monolayer. Based on these findings the cell culture was terminated after 3 days for all consecutive experiments.

**Figure 5:**
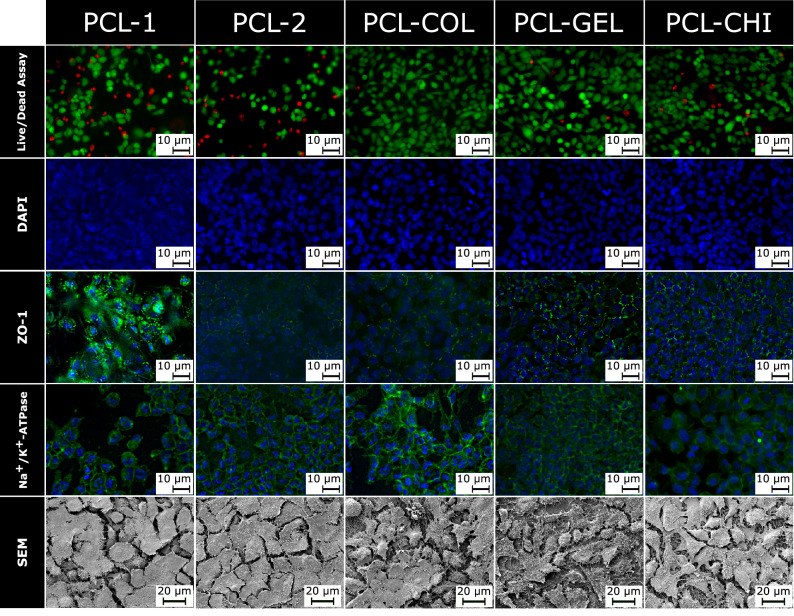
Staining of human corneal endothelial cells (HECEs) with fluorescein diacetate (FDA) and propidium iodide (PI) (top row) allows the distinction between live and dead cells. PCL-COL and PCL-GEL have a significantly higher cell viability (*p* < 0,001) compared to pure PCL scaffolds. Immunofluorescence staining with the antibodies ZO-1 (third row) and Na^+^/K^+^-ATPase (forth row) are indicators for working cell functions. SEM images (bottom row) indicate cell monolayers for all samples.

Cytotoxicity measurements revealed an overall non-toxic behaviour of the scaffolds. Images from the live/dead assay are shown in Fig. 5, first row. Cell viability for the PCL-1 and PCL-2 were 80 ± 14% and 77 ± 10%, respectively. A significant higher cell viability could be detected for the PCL-COL (97 ± 3%) and PCL-GEL (98 ± 2%) scaffold compared to the PCL-1 and PCL-2 scaffold (α < 0.05). Both scaffolds showed higher cell viability, than the positive control with 95 ± 5%. The PCL-CHI scaffold had a slightly reduced cytocompatibility with 91 ± 5%. In summary, all scaffolds showed sufficient cell viability, whereby the PCL composite scaffolds offered partially significant higher cell viability.

Immunofluorescence staining with antibodies against ZO-1 (zonula occludens-1, also known as tight junction protein-1) and Na^+^/K^+^-ATPase (the sodium–potassium ATPase is an enzyme from the class of transmembrane proteins that is anchored in the cell membrane. It catalyses the active transport, i.e. against the concentration gradient, of 3 sodium ions out of the cell and 2 potassium ions into the cell by hydrolysis of adenosine triphosphate (ATP) and thus functions as an antiporter) are displayed in Fig. 5, third and fourth row. As shown in the images for the PCL-1 scaffold, the fluorescence signal interfered with the nanofibers, resulting in a superposition of the scaffold structure and the cells. Nevertheless, expression of the applied antibodies could be detected. For all samples, ZO-1 was expressed at the cell borders and the typical hexagonal arrangement of the HCECs was displayed. HCEC pump sites were visualized by reactivity with the Na^+^/K^+^-ATPase antibody, indicating the presence of the protein in the in vitro experiments.

Cell surface morphology is shown in the SEM images, Fig. 5, bottom row. For the PCL-1 and PCL-2 scaffold, a smooth cell surface was visible. The cells were homogeneously distributed at the surface of the scaffold. Especially the cells on the PCL-2 scaffold showed an even distribution and thus forming a partially dense barrier. The cracks in between were presumable an artefact from the sample preparation or lacking cell–cell contact due to the short culture times. The blend scaffolds showed cells, not yet formed into the partially dense barrier as detected for the PCL-2 and PCL-1 scaffolds. Nevertheless, the cells were mostly flat and nicely spread upon the scaffold. PCL-CHI scaffolds revealed a cell–cell interaction similar to the pure PCL nanofibrous scaffold. Again, this similarity might be due to the low content of chitosan in the initial spinning solution.

**Figure 6:**
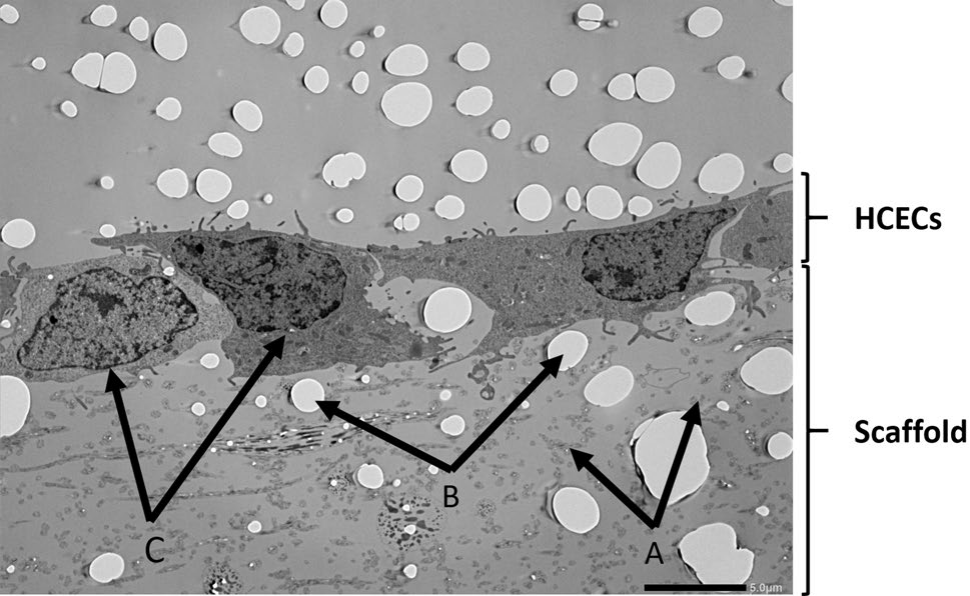
TEM image for the PCL-COL scaffold. (**A**) PCL-COL fibers (**B**) Artefacts from sample preparation (**C**) cell nucleus. Due to the low magnification single PCL-COL nanofibers are hardly visible. Scale bar: 5 µm.

In Fig. 6, an exemplary TEM image for the PCL-COL scaffold is shown. Fibers are exemplary indicated with arrows A. The white spots in the TEM image were flaws from the sample preparation, indicated by the arrows B, while arrows C point at the cell nuclei of neighboring cells. Due to the low contrast between polymeric nanofibers and embedding medium, the nanofibers are hardly visible in Fig. 6. Therefore, the scaffold as well as the HCECs are marked at the right margin. It can be seen, that some fibers were fully enclosed by pseudopods, while no further in-growth of the cells into the scaffold could be detected. The superficial pores were penetrated by pseudopods from the HECEs. However, cells were only interacting with the first two to three layers of nanofibers, with the HCECs placed on top of the scaffold, properly attached to them by superficial anchoring in the top layers of nanofibers. Similar findings could be observed for the other scaffolds and are shown in the supplementary information (Figs. S1, S2, S3 and S4).

## Discussion

In this study, we could demonstrate that PCL-based electrospun nanofiber scaffolds can be tissue-engineered with HCECs. Blending of PCL can be achieved with different naturally-derived components and by adapting the spinning parameters nanofibrous scaffolds can be manufactured. Using tissue carrier and self-made PTFE hollow cylinder, a feasible method was developed for the culture of cells on nanofibrous scaffolds from the electrospinning. Using hollow cylinders, a defined and reproducible amount of cells could be seeded onto the scaffolds, whereby systematic errors could be minimized. After removing the hollow cylinders, the cultivation of cells on the scaffolds may be continued without any boundary effects. By blending with natural polymers as collagen or gelatine, scaffolds with increased hydrophilic properties can be fabricated. FT-IR measurements confirm the blend morphology while TEM imaging reveals a phase separation between the PCL matrix and the blend. This phase separation is also visible in the PCL-CHI fibers. The cell viability of PCL-COL and PCL-GEL scaffolds is significantly higher, than that of PCL and PCL-CHI scaffolds. Electron microscopy and fluorescence staining showed a distinctive cell-scaffold interaction for all samples.

The fiber diameters produced in this study were comparable to the results from literature. Stafiej et al.^51,54^ obtained in their work similar fiber diameters for the PCL-2, PCL-1 as well as for the PCL-CHI scaffolds. In the case of the PCL-CHI scaffolds, the bimodal distribution of fiber diameter was observed, too. Similar to other studies, the solubility of chitosan in an adequate amount was not possible^54^. As preliminary experiments have shown, only a very low content of chitosan could be diluted in the spinning solution due to the hygroscopic nature of chitosan. Furthermore, higher amounts of chitosan are not feasible for electrospinning as batch-to-batch variations of natural derived chitosan and of unspecific specifications concerning molecular weight and degree of deacetylation have a considerable impact on the spinning solution. In a study by Dippold et al.^55^ PCL-COL fibers with similar results as shown in the present study were observed. The similarities in spinnability and resulting fiber morphologies for the PCL-COL and PCL-GEL blends from the same set-up and parameters allow the conclusion that technical aspects of electrospinning of PCL blended with collagen or gelatine resemble one another. The slightly smaller fiber diameter of the PCL-GEL fibers may arise from the less ordered structure of gelatine molecules compared to the complex structure of the triple helix of collagen, resulting in a marginal decreased viscosity and therefore in thinner fibers^52^. In a subsequent study, Dippold et al.^55^ revealed a significant batch-to-bath inconsistency for collagen which has not been found for the less ordered gelatine molecules in electrospinning processes.

To our knowledge nanofiber cross-sections from the electrospinning following in vitro cell culture experiments with corneal endothelial cells have not been studied to date. TEM images and results from FT-IR measurements indicate an incorporation of the blend phases into the PCL matrix. This and the effect on the contact angle were reported by many studies^47,50,51^. Typical peaks of the blend phases can be found in the FT-IR spectra while a reduced contact angle is measured. In comparison to the studies mentioned before, we measured the contact angle of film casted samples. In this way, surface characteristics arising from the structure of nanofibrous scaffolds are not taken into account. For example, Stafiej et al.^51^ measured a contact angle of 107.6 ± 2.5° for PCL-CHI nanofibrous scaffolds, significantly higher than the results reported in our study. This leads to the conclusion, that the nanoscaled structure of the scaffolds may yield higher apparent contact angles due to the microporous structure of the electrospun scaffolds.

While several studies evaluate the biocompatibility of nanofibrous matrices with corneal epithelial or stromal cells e.g.^51,56^ for corneal repair, corneal endothelial cells were less studied in this respect. Kruse et al.^16^ studied three different polymers, including PCL. It has to be mentioned, that due to a different spinning solution significantly different fiber diameters with 2.3 ± 0.1 µm were obtained. Initially, 400 cells per mm^2^ were seeded and after seven days the cell viability was evaluated without further characterising the ECD. Finally, a cell viability of 80.8 ± 3.3% was observed, similar to the results from our study. It seems, that fiber diameter only had a minor impact on the cell viability within the range of fiber diameter investigated compared to material characteristics, e.g. contact angle.

Staining of corneal endothelial cells regarding cell functionality has been widely studied for different carrier systems and antibodies e.g.^15,17,18,25,57^, whereby mostly naturally derived carriers were investigated. Kim et al.^17^ studied the cell-scaffold interactions for random and uniaxially aligned PCL and PCL-COL nanofibers. They reported similar results concerning the expression of ZO-1 and Na^+^/K^+^-ATPase antibodies. It is noteworthy to mention, that the cell morphology in the case of aligned nanofibers are rather elongated alongside the fiber orientation and no honeycomb structure, typically for HCECs could be observed. A further characterisation of the cell-scaffold interactions with transmission electron microscopy as shown in our study has not yet been carried out.

In further studies, a two-step culture medium approach should be evaluated. In our study, the proliferation capacity of the endothelial cells led to quite short culture periods. ECD on the scaffolds reached a cell density level comparable to the in vivo level after three to five days. Longer culture times resulted in undesired high cell densities and the loss of the HCEC typical conformation due to the ongoing proliferation of the immortalized cell line HCEC-12. A two-step culture medium approach as proposed by Peh et al.^58^ may overcome this issue. Another approach might be the use of primary cells instead of the immortalized HECE-12 cell line. Nevertheless, the current approach gives valuable information for a first screening of electrospun scaffolds and the evaluation of blend effects on the biocompatibility. Further studies should focus on functional studies, using donor corneas and artificial anterior chambers to simulate DMEK surgeries and check for cell functionalities ex vivo. For the clinical use, of course, scaffold thickness has to be reduced to a thickness 5–10 µm to increase light transmission through the scaffold giving potential patients an immediate benefit. In our study, we were able to reduce the scaffold’s thickness to a few micrometres. With decreasing scaffold thickness absorption and scattering of electromagnetic waves in the visible spectrum is minimized and consequently scaffold transparency is improved.

Taking into consideration that collagen fibrils in the human cornea are significantly smaller in diameter (25–35 nm^59^) compared to the produced nanofibers in this study, further efforts might be directed in the development of comparable fiber diameters. Thus, an exact copy of the ultrastructure of the native tissue can be investigated.

In conclusion, blending of PCL with collagen, gelatine or chitosan leads to nanofibrous scaffolds with fiber diameters of a few hundred nanometers. PCL-COL and PCL-GEL solutions enhance the properties of electrospun nanofibrous scaffolds for the use in lamellar keratoplasty. Both blends lead to increased cell viability compared to PCL and PCL-CHI scaffolds. For all tested samples, no cytotoxic threat could be detected while antibody staining showed working endothelial cells. Thus, electrospun PCL and PCL-blend scaffolds seem to be a reasonable approach as substitutes for posterior lamellar keratoplasty.

## Methods

### Fiber characterisation

Fiber morphology and diameter distribution were examined using scanning electron microscopy imaging. The samples were coated gold (Q150T Turbo-pumped Sputter Coater, Quorum Technologies Inc., Guelph, ON, CA) before mounting in the vacuum chamber of the SEM (CrossBeam Carl Zeiss Microscopy GmbH, Oberkochen, Germany). For every scaffold, 10 pictures were taken and in total the diameter of 200 fibers was measured using ImageJ software. FT-IR spectroscopy was performed for all five types of fibrous scaffolds using a Nicolet 6700 ATR-FTIR (Thermofisher Scientific, Waltham, MA, USA) in the wavenumber range from 4000–650 cm^−1^. Additionally, the contact angle of film casted samples was measured (OCA 20, DataPhysics Instruments GmbH, Filderstadt, Germany). Every measurement was performed ten times and the mean with standard deviation was calculated.

### Cell culture

Cell experiments were conducted using HCEC-B4G12, a subpopulation from the parental cell line HCEC-12 (Leibnitz Institute, Braunschweig, Germany). Cultivation of HCECs was performed in a mixture of HAM’s F12 and F199 culture medium (Lonza Group Ltd, Basel, Switzerland) supplemented with fetal bovine serum (15%, Gibco, Thermofisher Scientific, Waltham, MA, US), penicillin/streptomycin (1%), l-glutamine (2 mM), bFGF (2 ng/ml) and L-ascorbic acid 2-phosphate (0.3 mM, all purchased from Sigma Aldrich, Saint Louis, MO, USA)^57^. Cells were kept in an incubator at 37 °C, 5% CO_2_ and 21% O_2_. HECEs were cultured in cell culture flasks (Greiner Bio-One International GmbH, Germany) until 80–90% confluency before the initial cell seeding.

The nanofibrous scaffolds were clamped into MINUSHEET® tissue carrier (Minucells and Minutissue, Bad Abbach, Germany) and placed in a 24-well plate. Before cell seeding, the scaffolds were washed for 30 min in ethanol (70%) and rinsed with PBS three times afterwards. Then, the scaffolds were incubated in 1 ml of medium and stored in the incubator for at least 12 h. On each scaffold, 20,000 cells were seeded yielding an initial cell density of about 700 cells per mm^2^. The PTFE cylinder was removed at the earliest 6 h after cell seeding. Medium was replaced every second day, starting at day 1.

### Cell morphology

After three days of cultivation, the scaffolds with the cells were rinsed with PBS three times and thereafter fixed in Ito's fixative (2.5% glutaraldehyde, 2.5% paraformaldehyde and 0.3% picric acid dissolved in PBS, pH 7.3). In the next step, the scaffolds were washed with sodium cacodylate buffer and dehydrated in alcohol and acetone. Afterwards, samples were dried with an EM CPD300 Critical Point Dryer (Leica, Wetzlar, Germany) and sputtered with gold (Low Vacuum Coater EM ACE200, Leica, Wetzlar, Germany). Eventually, the scaffolds were analysed using a scanning electron microscope (REM JSM-IT300LV, JEOL GmbH, Freising, Germany). For the TEM sample preparation, scaffolds were fixed after three days of in vitro culture in Ito's fixative for 30 min and then embedded in Epon (Carl Roth, Karlsruhe, Germany). Semithin sagittal sections were created with a microtome (Ultracut E; Reichert Jung, Vienna, Austria). The semithin sections were then stained with uranyl acetate and lead citrate. Transmission electron microscopy imaging was performed using a JEM-1400 Plus (JEOL GmbH, Freising, Germany).

### Proliferation analysis

Scaffolds with HCECs were rinsed with PBS three times and fixed in methanol at − 20 °C for 5 min together with the scaffolds. The samples were then stained with hematoxylin and eosin (HE) and embedded with Aquatex mounting medium (Merck KGaA, Darmstadt, Germany) between a glass slide and a cover slip. Samples were evaluated using a Keyence BZ9000 microscope (Keyence GmbH, Neu-Isenburg, Germany). Cell density was assessed by counting the cell number within a particular area using ImageJ software and calculating the respective cell density. For each material and period of culture, six scaffolds were evaluated.

### Cell viability assay

Examination of scaffold toxicity was evaluated using fluorescein diacetate (FDA) and propidium iodide (PI) (Sigma Aldrich, Saint Louis, MO, USA) according to the supplier. Cell viability was examined using the fluorescence microscope Keyence BZ9000 (Keyence GmbH, Neu-Isenburg, Germany). Every material was tested in triplets and for each scaffold three randomly selected microscopic details were analysed. As positive control, cells were seeded on standard 24-well-plates (Greiner Bio-One International GmbH, Germany). As negative control, DMSO (0.1 ml) was added to the culture medium 24 h before the cell viability assay was conducted.

### Immunohistochemistry

HCECs were cultured on the scaffolds for three days before the cells were fixed using methanol at − 20 °C for five minutes. Scaffolds were thoroughly rinsed with PBS and excess binding sites were blocked with 10% nonfat milk/PBST (1 mL Tween 20/1 L 1 × PBS) at room temperature for 1 h. Then, the primary antibodies were applied over night at 4 °C. Immunofluorescence staining was performed with antibodies ZO-1 (1:200, Sigma Aldrich, Saint Louis, MO, USA) and Na^+^/K^+^-ATPase (1:100, Invitrogen, Carlsbad, CA, USA). Afterwards, the secondary antibody (1:1000, Alexa488, MoBiTec GmbH, Göttingen, Germany) was incubated for 2 h at room temperature and nuclei staining was done using 4′,6-Diamin-2-phenylindol (DAPI, 1:1000, Sigma Aldrich, Saint Louis, MO, USA) for 10 min at room temperature. Eventually, the scaffolds were fixed between a glass slide and coverslip using Dako Fluorescence Mounting Medium (Agilent Technologies, Santa Clara, CA, USA). The scaffolds were examined with a Keyence BZ9000 microscope (Keyence GmbH, Neu-Isenburg, Germany).

## Supplementary Information

Supplementary Information.

## Data Availability

The datasets generated during and/or analysed during the current study are available from the corresponding author on reasonable request.
