# Supplementary material for: Optimization of polycaprolactone - based nanofiber matrices for the cultivation of corneal endothelial cells
Source: Sci Rep. 2021 Sep 22;11:18858. doi: 10.1038/s41598-021-98426-6 (PMC8458296; doi:10.1038/s41598-021-98426-6)
Supplement: Supplementary file 1 — Supplementary Information. [file 41598_2021_98426_MOESM1_ESM.pdf]

Optimization of Polycaprolactone - based Nanofiber Matrices for the Cultivation of Corneal Endothelial Cells

Marcus Himmler, Fabian Garreis, Friedrich Paulsen, Dirk W. Schubert, Thomas A. Fuchsluger

Supplementary Information

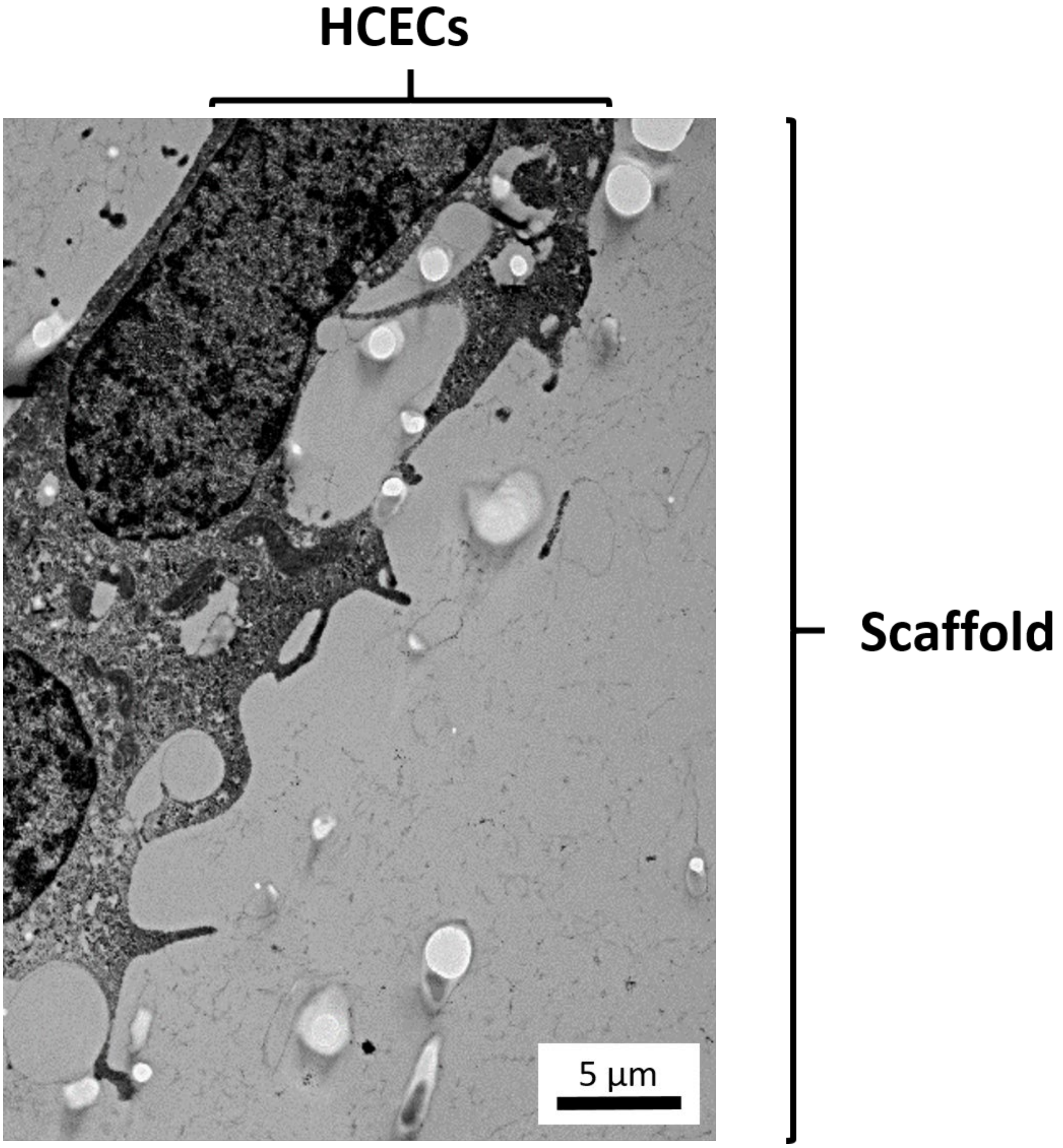

Figure S1: TEM image for the PCL-1 scaffold. HCECs and the scaffold are indicated at the margins.

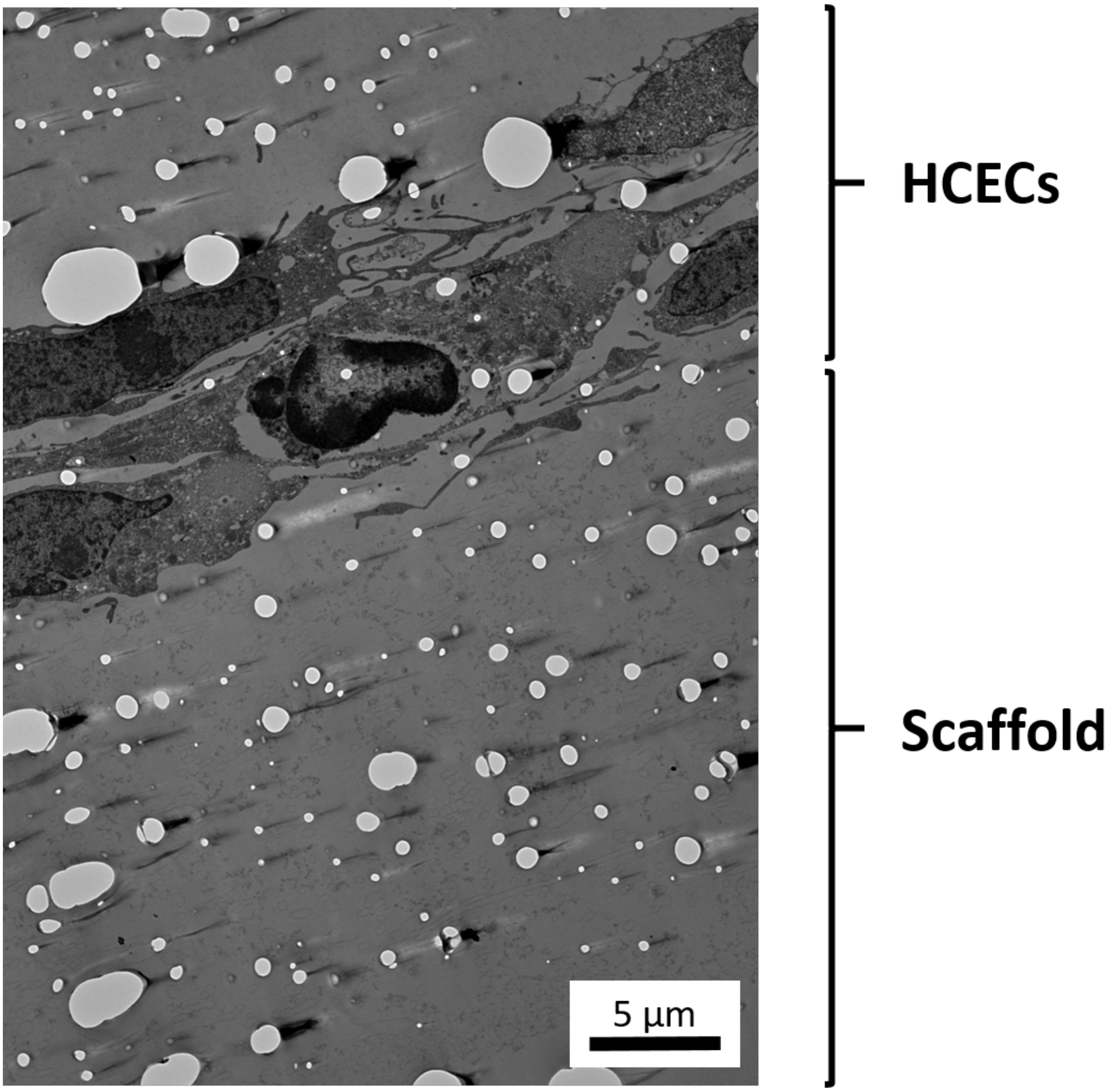

Figure S2: TEM image for the PCL-2 scaffold. HCECs and the scaffold are indicated at the margin.

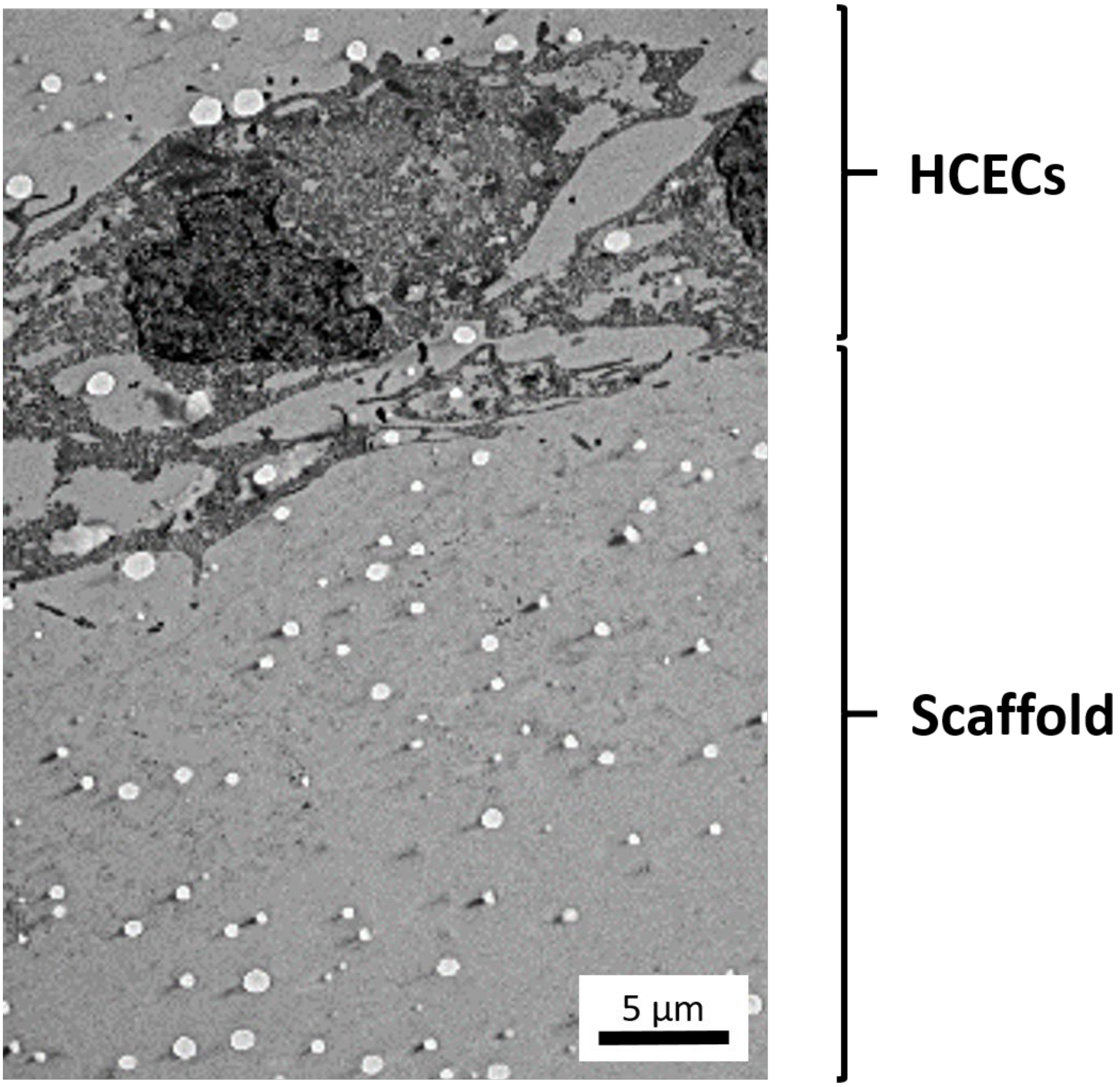

Figure S3: TEM image for the PCL-GEL scaffold. HCECs and the scaffold are indicated at the margin.

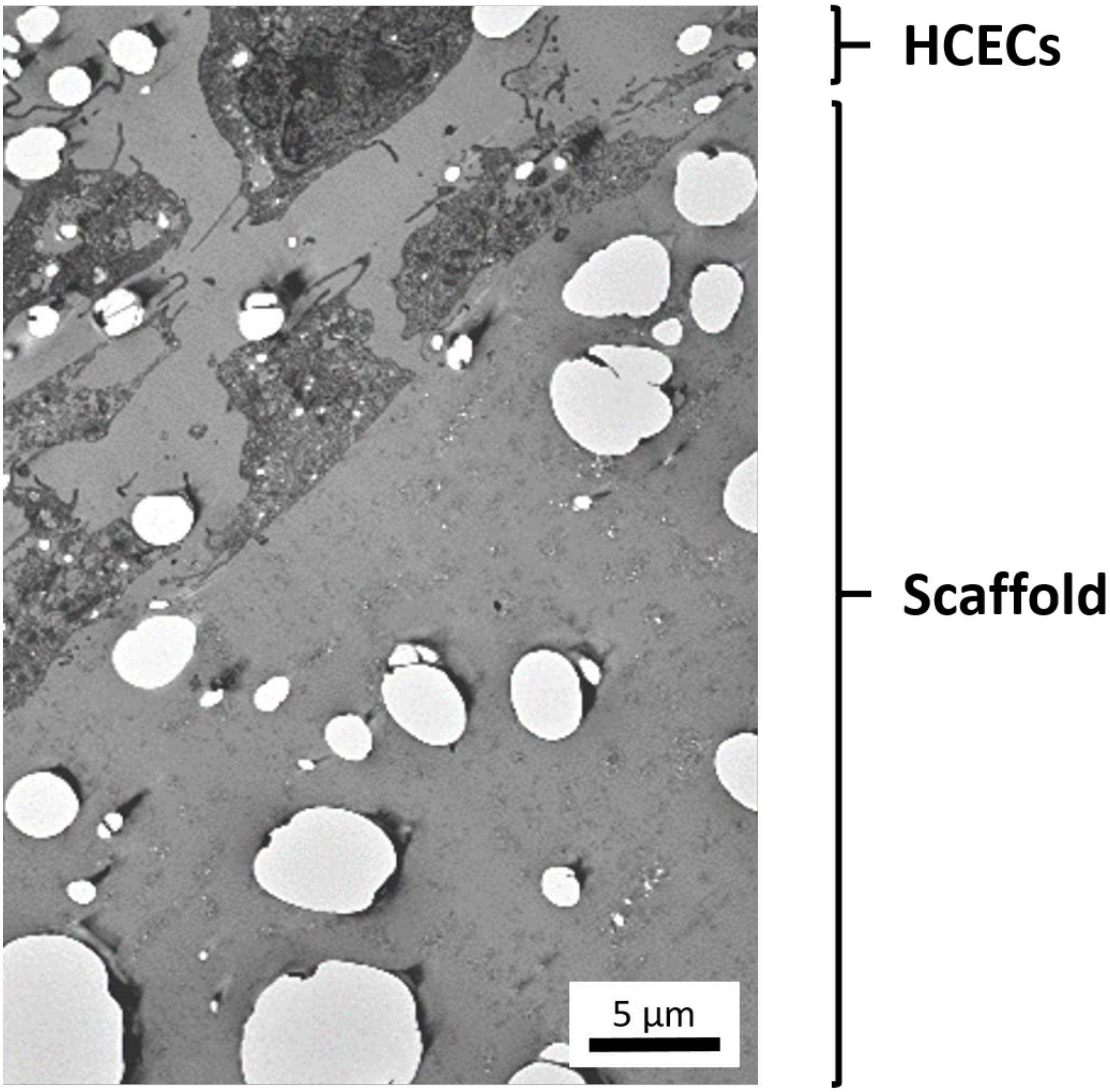

Figure S4: TEM image for the PCL-CHI scaffold. HCECs and the scaffold are indicated at the margin.
